# Electromyographic Analysis of Masticatory Muscles in Cleft Lip and Palate Children with Pain-Related Temporomandibular Disorders

**DOI:** 10.1155/2018/4182843

**Published:** 2018-05-13

**Authors:** Liliana Szyszka-Sommerfeld, Teresa Matthews-Brzozowska, Beata Kawala, Marcin Mikulewicz, Monika Machoy, Włodzimierz Więckiewicz, Krzysztof Woźniak

**Affiliations:** ^1^Department of Orthodontics, Pomeranian Medical University of Szczecin, Al. Powstańców Wlkp. 72, Szczecin 70111, Poland; ^2^Department and Clinic of Maxillofacial Orthopaedics and Orthodontics, Poznan University of Medical Sciences, 70 Bukowska Street, Poznań 60812, Poland; ^3^Department of Maxillofacial Orthopaedics and Orthodontics, Wroclaw Medical University, 26 Krakowska Street, Wrocław 50425, Poland; ^4^Department of Maxillofacial Orthopaedics and Orthodontics, Division of Facial Abnormalities, Wroclaw Medical University, 26 Krakowska Street, Wrocław 50425, Poland; ^5^Department of Prosthetic Dentistry, Wroclaw Medical University, 26 Krakowska Street, Wrocław 50425, Poland

## Abstract

**Aim:**

The aim of this study was to assess the electrical activity of temporalis and masseter muscles in children with cleft lip and palate (CLP) and pain-related temporomandibular disorders (TMD-P).

**Methods:**

The sample consisted of 31 CLP patients with a TMD-P (mean age 9.5 ± 1.8 years) and 32 CLP subjects with no TMD (mean age 9.2 ± 1.7 years). The children were assessed for the presence of temporomandibular disorders (TMD) using Axis I of the Research Diagnostic Criteria for TMD (RDC/TMD). Electromyographical (EMG) recordings were performed using a DAB-Bluetooth Instrument (Zebris Medical GmbH, Germany) in the mandibular rest position and during maximum voluntary contraction (MVC).

**Results:**

The rest activity of the temporalis and masseter muscles was significantly higher in TMD-P group compared with non-TMD children. A significant decrease in temporalis muscle activity during MVC was observed in TMD-P patients. There was a significant increase in the Asymmetry Index for temporalis and masseter muscle rest activity in the TMD-P group.

**Conclusion:**

Cleft children diagnosed with TMD-P have altered masticatory muscle activity, and this can affect their muscle function.

## 1. Introduction

Cleft lip and palate (CLP) is one of the most common congenital deformities of the craniofacial area requiring long-term functional and aesthetic rehabilitation [1]. Complete clefts of the lip and/or palate are immediately recognizable disruptions of normal facial structure [2]. In addition to dysfunctional facial expressions, patients with CLP may have serious functional problems with sucking, swallowing, breathing, chewing, speaking, hearing, and social integration [3, 4].

The prevalence of malocclusions in CLP patients is relatively high [5]. The most common occlusal disorders in patients with clefts are crossbites and class III malocclusions [6, 7]. Malocclusions, particularly of the transverse type where disrupted symmetry of the dental arches can be clinically observed, are a potential cause of functional disorders of the stomatognathic system [8]. Hence, patients with CLPs are potentially at risk of developing temporomandibular disorders (TMD), due to psychosocial burdens and malocclusions predisposing them to this condition [6, 9]. This is consistent with previous reports [10–12]. The importance of a patient's lower socioeconomic status as a likely important factor in the development of TMD in CLP patients has also been noted [13].

Temporomandibular disorders (TMD) are a collective term embracing a number of clinical problems affecting the masticatory muscles, the temporomandibular joint (TMJ), and associated structures [14]. Pain-related temporomandibular disorders (TMD-P) are the most prevalent conditions among TMD [15]. They comprise myalgia, arthralgia, and headaches attributed to TMD [16]. The primary manifestations of TMD-P are pain of a persistent, recurring, or chronic nature in the masticatory muscles, TMJ, or in adjacent structures [17, 18]. The other major symptoms include limitation in the range of mandibular motion and joint noises [14, 17]. The pain may radiate to different regions, such as the dental arches, ears, temples, forehead, occiput, and the cervical region of spine or shoulder girdle [18]. The aetiology of pain-related TMD is considered to be multifactorial and to result from a complex interaction between biological, psychological, social, and environmental variables [19, 20].

The prevalence of TMD signs and symptoms in children and adolescents in the general population ranges from 1 to 50%, and TMD-P from 1% to 22% [21–26]. The prevalence of objective and subjective symptoms of TMD in children with CLP is relatively higher [13].

Surface electromyography (sEMG) is the study of muscle function based on an analysis of the electrical signals produced during muscular contraction. The sEMG method is painless and innocuous, and these are important factors when conducting studies involving children [27, 28]. It has been widely used in research settings for the assessment and follow-up of patients with TMD [29, 30], and numerous studies have demonstrated altered electromyographical (EMG) values in the masticatory muscles of patients with TMDs [31–34]. Subjects diagnosed with TMD-P alter the recruitment of their jaw muscles [35]. Free nerve endings act as nociceptors activated by noxious stimulation such as temporomandibular joint (TMJ) overloads and/or masticatory muscle ischemia, if it is prolonged and associates with muscle contractions [36, 37]. A correlation has been observed between a decrease in the motor unit firing rate and muscle pain intensity, although the central mechanisms involved remain unclear [38]. Maximum EMG activity is greater in pain-free subjects than in patients with pain-related TMD [33, 39]. Nevertheless, to the authors' knowledge, until now there have been no EMG studies on masticatory muscle activity in cleft lip and palate subjects with a TMD-pain diagnosis. The identification of the electromyographic pattern of the mastication muscles is necessary in order to achieve functional improvement in the stomatognathic system, particularly in cleft children. For these reasons, it is essential to determine temporalis and masseter muscle activity in cleft lip and palate children with pain-related TMD by means of electromyography (EMG).

The aim of this study was to determine whether the electrical activity of temporalis and masseter muscles in children with complete CLP and pain-related TMD differs from that observed in CLP individuals with no TMD. The null hypothesis was that there are no differences between CLP individuals with TMD-P and non-TMD with regard to the electrical activity of the temporalis and masseter muscles in the mandibular rest position and during maximum voluntary teeth clenching.

## 2. Material and Methods

The clinical research was registered as a case-control study in the ClinicalTrials.gov database and assigned the number NCT03308266.

### 2.1. Study Sample

The sample comprised 63 children with cleft lip and palate and mixed dentition. In accordance with the outcomes of Axis I of the Research Diagnostic Criteria for Temporomandibular Disorders (RDC/TMD) [17], the children were divided into two groups: a TMD-pain group and a non-TMD group. The groups were matched for age and gender. The TMD-pain group included 31 children (15 girls and 16 boys) aged between 6.4 and 13.9 (mean 9.5 ± 1.8) with complete cleft lip and palate and a pain-related TMD diagnosis. The control group consisted of 32 subjects (14 girls and 18 boys) aged between 6.7 and 11.7 years (mean 9.2 ± 1.7) with CLP and no TMD diagnosis. The subjects were selected from a total of 90 patients who had been referred to three Cleft Care Centres in Szczecin, Poznań, and Wrocław, Poland, between November and December 2015. All had undergone lip and palate surgery at one of four different Plastic Surgery Clinics in Poland according to the following protocols: A. a two-stage lip and palate repair procedure, that is, a lip operation at the age of 3–6 months, followed by palate closure (hard and soft palate in one-step procedure) at the age of approximately 12 months; B. single-stage lip and palate repair in children at the age of about 6 months, that is, the lip and hard and soft palate were closed in a single operation. The application of the adopted inclusion and exclusion criteria resulted in 27 of the subjects being excluded from the study and 63 of the participants qualifying for further examination. The inclusion criteria for the TMD-pain group were as follows: meeting Axis I of the RDC/TMD diagnosis criteria with pain (arthrogenous or myogenous TMD), children of both sexes with mixed dentition, undergoing lip and palate surgery, the presence of a cleft lip and palate without a syndrome, a sequence or karyotype abnormalities, and consent to participate voluntarily in the study. The inclusion criteria for control group were as follows: children without any TMD diagnosis according to the RDC/TMD protocol, children of both sexes with mixed dentition, undergoing lip and palate surgery, the presence of a cleft lip and palate without a syndrome, a sequence, or karyotype abnormalities, and consent to participate voluntarily in the study. The exclusion criteria for both groups included: meeting Axis I of the RDC/TMD diagnosis criteria without pain, the presence of systemic or rheumatologic diseases, a history of cervical spine or temporomandibular joint (TMJ) surgery, trauma or malformations, and completed orthodontic or masticatory motor system dysfunction treatment.

Masticatory motor system function was assessed on the basis of a clinical examination and electromyographic procedures.

### 2.2. Clinical Examination

Anamnestic interviews were conducted, which covered the patients' general medical history and provided detailed information on the patients' masticatory motor systems, including subjective TMD symptoms, such as jaw pain during function, frequent headaches, jaw stiffness/fatigue, difficulty in opening the mouth wide, teeth grinding, and TMJ sounds. The children were assessed for the presence of temporomandibular disorders using Axis I of the Research Diagnostic Criteria for TMD (RDC/TMD) by a single trained examiner. This helped ensure standardized procedures for epidemiological studies, unified TMD diagnostic and exploratory criteria, and a comparison with the results of other similar studies [17, 40]. The clinical signs were assessed using the RDC/TMD criteria, including pain on palpation, mandibular range of motion (mm), associated pain (jaw opening pattern, unassisted opening, maximum assisted opening, mandibular excursive, and protrusive movements), sounds coming from the TMJ, and tenderness induced by muscle and joint palpation. Generally, the RDC/TMD criteria classify forms of TMD into three diagnostic categories:

- Group I: muscle disorders (Group Ia with myofascial pain and Group Ib with myofascial pain with limited opening);
- Group II: disc displacement (Group IIa with reduction, Group IIb without reduction with limited opening, and Group IIc without reduction but without limited opening);
- Group III: arthralgia (Group IIIa) or arthritis (Group IIIb/IIIc).

RDC/TMD specifies distinct operational criteria for each TMD subtype; for example, a myalgia diagnosis is made if a person reports pain in the face or mastication muscles at rest or during function, as well as the presence of pain upon palpation at 3 or more sites. An arthralgia diagnosis includes pain upon palpation of the TMJ and joint-related pain during movements of the opening mouth, mandibular excursive, and protrusive movements; a diagnosis of arthritis includes pain in addition to reported clicking sounds upon palpation. Thus, every TMD subject could have both a masticatory muscle pain diagnosis and/or a TMJ pain diagnosis.

Replicate measurements of clinical signs of TMD were recorded for twenty randomly selected children in order to assess intraexaminer reliability. For this purpose, intraclass correlation coefficients (ICCs) were calculated for both continuous and dichotomous variables of the RDC/TMD examination. The considered ICC values were as follows: ICC < 0.4 which corresponds to poor reliability; 0.4 ≤ ICC ≤ 0.75—fair to good reliability; ICC > 0.75—excellent reliability [41, 42].

The intraoral examination included an analysis of the dental arch shape on three planes together with a reciprocal analysis of both dental arches. The following occlusal characteristics were evaluated: sagittal relationship of the permanent first molar according to Angle's classification, posterior crossbite, overbite, overjet, and lateral open bite.

### 2.3. Electromyographical Examination

We followed the methods by Szyszka-Sommerfeld et al. [43]. The EMG recordings were taken with a DAB-Bluetooth Instrument (Zebris Medical GmbH, Germany) by a single experienced researcher. During the recordings, each patient sat in a comfortable chair without a head support and was instructed to assume a natural head position [44]. This position allows us to eliminate or limit any unintentional movements from other parts of the body.

Surface EMG signals were detected with four silver/silver chloride (Ag/AgCl), disposable, self-adhesive, bipolar electrodes (Naroxon Dual Electrode, Naroxon, USA) with a fixed interelectrode distance of 20 mm. The electrodes were precisely positioned on the anterior temporalis muscle and the superficial masseter on both the left and the right sides parallel to the muscular fibres. The placement of the electrodes was exactly the same as previously described by Ferrario et al. [45] The temporalis anterior muscle: vertically along the anterior margin of the muscle; the masseter muscle: parallel to the muscular fibres with the upper pole of the electrode located at the intersection between the tragus-labial commissura and exocanthion-gonion lines. A reference electrode was applied inferior and posterior to the right ear.

The surface of the patient's skin was cleaned of impurities and degreased with a 70% ethyl alcohol solution by wiping it several times with disposable cotton wool. Slight reddening of the skin after cleaning is a clinical indicator that the site has been properly prepared. To confirm that the tested area had been properly prepared, an impedance test was performed with Metex P-10 a measuring device (Metex Instruments Corporation, Korea) with an accuracy of 2%. This device measures the resistance between a pair of electrodes for a period of 5 minutes from the placing of the electrodes. Further examinations would be conducted if the test produced a positive result (low skin tissue impedance).

The EMG recordings were taken 5 minutes later. The electrical activity of the temporalis and masseter muscles was then measured during the course of three different tests:

1. Rest activity of the masticatory muscles in the clinical rest position;
2. Maximum voluntary clench (MVC) in the intercuspal position where the patient was asked to clench as hard as possible for 5 seconds;
3. Maximum voluntary clench (MVC) with two 10-mm thick cotton rolls placed on the mandibular second premolars and molars or on the mandibular second milk molars and the first permanent molars and the patient was asked to clench as hard as possible for 5 seconds.

To avoid any effects of fatigue, a rest period of at least 5 minutes was allowed between each of these recordings. The EMG recordings were repeated at least three times to ascertain stability. The first recordings were eliminated as a “learning” sequence since they were frequently observed to be dissimilar to the other two repetitions. In a single subject, all EMG data were the arithmetic means of these last two surface EMG recordings. The patients were allowed to relax for 1 minute between each activity.

The DAB-Bluetooth Instrument was interfaced with a computer, which presented the data graphically and recorded it for further analysis. The EMG signals were amplified, digitized, and digitally filtered. The basic component of the data analysis was the normalization process. Normalization involved referring the raw results (the mean values of the EMG potentials) to the data obtained from each patient after clenching on two cotton rolls (reference values) according to the following formula: mean values (*µ*V) during rest position or MVC/mean values (*µ*V) during MVC with two 10 mm cotton rolls × 100%. For each muscle, the EMG potentials were expressed as a percentage of the MVC value using cotton rolls (unit *μ*V/*μ*V%). This procedure was essential for the preliminary processing of raw data to ensure intercomparisons and further analysis. In order to compare EMG recordings among different subjects, it was necessary to relate all measurements to the electrical muscle activity detected during certain standardization recordings, such as MVC. The EMG potentials collected in MVC are reported to have the highest repeatability. Among the various protocols, MVC on cotton rolls is reported to have the lowest interindividual variability, and a method based on this standardization is now commonly used [45–48]. According to this protocol, normalized EMG data will provide information on the impact of occlusion (teeth contact) on neuromuscular activity, while avoiding individual variability (anatomical variations, physiological and psychological status, etc.) and technical variations (muscle cross-talk, electrode position, skin and electrode impedance, etc.).

Finally, the Asymmetry Index (As, unit %) was recorded to assess asymmetry between the activity of the left and right jaw muscles according to a formula ranging from 0% (total symmetry) to 100% (total asymmetry) [49].

(1) $$\begin{array}{c} \text{As}=\frac{\sum_{i=1}^{N}\left|R_{i}-L_{i}\right|}{\sum_{i=1}^{N}\left(R_{i}+L_{i}\right)}\cdot 100 \end{array}$$

To investigate the repeatability of the recording protocol, duplicate EMG evaluations were performed on the 20 subjects by the same operator, after a gap of 15 minutes between the two recordings. We asked the subjects to remain relaxed during this 15-minute break once the electrodes had been removed from their muscles and to walk around the laboratory if they needed to. The results of the first and second set of experiments demonstrated the repeatability of the EMG measurements. The data were presented as the mean values of the electrical activity of the temporalis and masseter muscles in rest position and during MVC. The repeatability of electrode positioning was maintained by using a standard procedure for positioning the electrodes. To assure standard results during the EMG examination, the electrodes were placed accurately at the area of muscle belly contraction [50].

### 2.4. Statistical Analysis

The homogeneity of variance was evaluated using the Levene test. The normality test applied was the Kolmogorov–Smirnov test. The results of the EMG recordings and the repeatability of the EMG measurements were analysed using Student's *t*-test and the Mann–Whitney *U* test to determine differences between the mean values of the independent variables. The chi-square test was used to determine differences in the prevalence of malocclusions between the groups of participants. The level of significance was set at *P*=0.05.

## 3. Results

The reliability value for the RDC/TMD clinical examination ranged from good to excellent (from 0.62 to 1.0).  Table 1 presents the distribution of TMD-P subjects according to their RDC/TMD diagnosis. Myofascial pain with no limited mouth opening (Group Ia) and arthralgia (Group IIIa) were diagnosed in 38.7% of the TMD-P patients, while 9.7% of the children were diagnosed with myofascial pain with limited mouth opening (Group Ib) and 12.9% of the subjects received a mixed TMD-pain diagnosis (Groups Ia and IIIa or Ib and IIIa).

The occlusal characteristics for both groups of children are presented in Table 2. There were no significant differences between TMD-pain group and control subjects in terms of the prevalence of malocclusions (*P* > 0.05).

Table 3 shows the results of the repeatability of the recording protocol. The differences between first and second evaluations of the electrical potentials of the masticatory muscles were not statistically significant in the case of any of the aforementioned activities (*P* > 0.05).

Analysis of the EMG recordings showed that the rest activity of both the temporalis and the masseter muscles was higher in subjects with CLP and TMD-P compared with non-TMD subjects (for temporalis muscles, *P*=0.0102; for masseter muscles, *P*=0.0188) (Table 4).

A significant increase was observed in the Asymmetry Index in relation to the rest activity of the temporalis (*P*=0.0218) and masseter muscles (*P*=0.0010) in patients with TMD-P compared with non-TMD children (Table 4).

Temporalis muscle activity during MVC was significantly lower in children from the TMD-pain group compared with children with no TMD (*P*=0.0477). There were no significant differences in masseter muscle activity during MVC between the TMD-pain group and control subjects (*P*=0.3163) (Table 5).

There were no differences between TMD-P and non-TMD subjects in terms of the Asymmetry Index for the temporalis and masseter muscles during MVC (temporalis muscle *P*=0.0858, masseter muscle *P*=0.0773) (Table 5).

In the rest position, differences between girls and boys in the TMD-pain and control groups with regard to the Asymmetry Index of the masseter muscles were statistically significant (for girls-*P*=0.0080 and for boys-*P*=0.0478) (Table 4). During MVC, a significant difference was observed in the Asymmetry Index of the masseter muscles between girls from the TMD-pain and the non-TMD group (*P*=0.0330) (Table 5).

## 4. Discussion

The present clinical study was designed to evaluate the electrical potentials of the masticatory muscles in cleft lip and palate children with pain-related temporomandibular disorders. Muscle activity was analysed in the mandibular rest position and during maximum isometric contraction (MVC). The EMG recordings showed that children diagnosed with CLP and pain-related TMD have greater temporalis and masseter muscle activity at rest, reduced temporalis muscle activity during maximum voluntary contraction, and a higher Asymmetry Index for the temporalis and masseter muscles in the rest position.

We used surface electrodes in this examination. They have the advantage of being noninvasive and for this reason are better tolerated by patients [51]. The repetition of the main experiment confirmed the repeatability of electrode positioning, as well as the entire protocol. No study on method error was performed with regard to the positioning of subjects in the “natural head position”. On the other hand, we employed a method that is considered one of the most repeatable, especially in adults (data about children are not so clear).

The study revealed hyperfunction of both temporalis and masseter muscles at rest position in patients with CLP and TMD-pain diagnoses. This means that, at rest, the EMG activity of the masticatory muscles was higher in CLP children with pain-related TMD than in subjects with no TMD. This behaviour may be explained by the need for greater muscle recruitment in individuals with TMD and pain when the mandible is at rest [52, 53]. This is probably due to sensorial-motor interactions, of which pain can modify the generation of action potentials and, eventually, myoelectric activity [54]. Riise [55] found that the activity of the temporalis muscle in the rest position was higher when occlusal interferences existed. In the long term, hyperactivity may be followed by structural adaptations, such as tooth movement, muscular reactions, and remodelling of the temporomandibular joints or it could lead to pathologic changes in the masticatory system.

Reduced temporalis activity in the cleft group diagnosed with TMD-pain during MVC suggests that there is an alteration in masticatory muscle recruitment compared to children without TMD. These alterations may be employed as an effective protective mechanism for damaged TMJs [56]. The specific recruitment of the temporalis muscle appears to be the result of descending central modulation subsequent to nociceptive stimuli of the affected TMJ and/or myofascial and/or periodontal nociceptors [36].

A higher Asymmetry Index for the temporalis and masseter muscles at rest in the TMD-P group indicates differential left-right muscle activity. Moreover, the increased Asymmetry Index in TMD-P patients may confirm a higher frequency of unilateral TMD in this group.

The results of the study indicate that in comparison to non-TMD cleft patients children diagnosed with CLP and TMD-P have altered masticatory muscle activity. As mentioned earlier, CLPs are strongly associated with the presence of malocclusions [5], which in turn could be a potential cause of TMD and can affect electrical muscle activity [8, 11, 12, 57]. Nevertheless, the prevalence of malocclusions in this study was similar in both children with TMD-P and in non-TMD children. However, other malocclusion-related factors, for example, the severity of malocclusion, were not determined. Further research would be needed to explore the association between TMD and muscle EMG activity in CLP subjects including factors that may contribute to TMD problems and changes in EMG pattern (e.g., malocclusion).

It is important to note that the children who participated in the study were still in the developmental stage. The alterations in masticatory muscle electrical activity showed by EMG recordings in children with TMD-P affect their muscle function. The altered muscle function in a growing stomatognathic system can result in malocclusion, or in the exacerbation of an already existing malocclusion, and this will be a significant risk factor promoting the development or progression of TMD problems in the future. Early investigation of the electromyographic characteristics of children could facilitate the development of treatment strategies aimed at normalizing muscle activity so as to achieve functional improvement in these patients. Of course, further studies that could be repeated for the same group of children in the future would provide evidence for a validity of EMG on the progression of their TMD.

This is the first report concerning masticatory muscle activity in children diagnosed with CLP and TMD-P based on the RDC/TMD criteria. As there have been no similar studies, it is difficult to compare our results with others. Nevertheless, the data obtained in our study could be referred to Li et al. [12], who evaluated masticatory muscle activity in patients with unilateral cleft lip and palate and anterior crossbite. The examined group included 29 individuals with CLP ranging in age from 11 to 21 years. Among them, 22 cleft patients had one or more symptoms of TMD on clinical examination. The control group consisted of 28 volunteers with no cleft abnormalities and normal occlusion. They found that compared to noncleft controls, patients with unilateral CLP had the following parameters for temporalis and masseter muscles: higher activation levels in the rest position, lower activity recorded during maximum clenching in the intercuspid position, and a higher Asymmetry Index.

An analysis of masticatory muscle EMG activity in children with TMD was the subject conducted by Chaves et al. [58]. They assessed the EMG activity of the masseter, temporalis, and suprahyoid muscles in 34 children aged 8–12 years: 17 children with TMD and 17 without TMD. The results of this study demonstrated a lower mean electromyographic ratio for masseter muscles and anterior temporalis muscles (sEMG-M/AT ratio) during maximum voluntary clenching in the TMD group. These results can be explained by three factors acting together: the lower mean raw activity of the masseter muscle compared to the anterior temporalis muscle in the TMD children, the lower mean raw activity of the masseter muscle in the TMD group compared to the control group, and the higher mean raw activity of the anterior temporalis muscle in the TMD group compared to the non-TMD group.

The relationship between TMD and TMD-P and the electrical activity of the masticatory muscles in adult females has been described by Rodrigues et al. [54] and Berni et al. [33]. They analysed the EMG potentials of the anterior temporalis, masseter, and suprahyoid muscles at rest position and during MVC on parafilm. They both found that EMG activity of the masticatory muscles at rest was higher in a TMD group than in a control group with no TMD. Rodrigues et al. [54] observed no differences with regard to MVC between such groups. However, Berni et al. [33] reported significantly lower activity in the masseter muscle. Similarly, Tartaglia et al. [34] and Liu et al. [59] found that temporalis and masseter muscle activity was significantly lower in TMD subjects than in non-TMD patients during MVC. Moreover, Liu et al. [59] observed greater EMG activity at rest position in the anterior temporalis muscle.

Khawaja et al. [15] assessed associations between masticatory muscle activity levels both when awake and during sleep among pain-related TMD diagnostic groups. Twenty-six adult subjects were classified into those diagnosed with TMD-P (myalgia and arthralgia) and those who were diagnosed with no pain. The data suggest a tendency towards increased masseter muscle activity in the TMD-pain group both when awake and during sleep. However, the same tendency was not noted in the temporalis muscle. They observed that temporalis muscle activity was only found to be higher in the pain-related TMD diagnoses group at extreme activity levels (<25% and ≥80% ranges).

The importance of such a parameter as muscular symmetry was noted by Liu et al. [59] and Tartaglia et al. [34]. Tartaglia et al. [34] found that symmetry in the temporalis muscles was greater in the control group than in TMD patients. Liu et al. [59] reported that asymmetry of the masseter muscle during MVC was significantly pronounced in TMD patients compared to normal subjects. The asymmetry of the anterior temporalis muscle was more pronounced in TMD patients during 70% MVC and was estimated at 28.6%, compared with 19.6% in normal subjects.

The results of the aforementioned studies suggest that an association exists between TMD and pain-related TMD and masticatory muscle EMG activity. Our study also revealed the influence of TMD-P on masticatory muscle EMG potentials in children with cleft lip and palate. Cleft children diagnosed with TMD-P have altered temporalis and masseter muscle activity compared with non-TMD subjects. From a clinical point of view, what is important is the fact that alteration of the pattern of muscle electrical activity in TMD-P patients can affect muscle fatigue, and can, as a consequence, have an impact on every function they perform in the stomatognathic system [60]. Such knowledge is essential when it comes to developing treatment protocols to normalize muscle activity and improve muscle function in these patients. However, we ought to be aware of the study's limitations, such as the relatively small number of subjects involved. In addition, the groups studied cover a comparatively wide age range. Hence, some differences between patients may result from variations in neuromuscular system development. Another possible limitation of the study might be the fact that the TMD-pain group included both joint- and muscle-related pain disorders, since EMG activity may vary in these subgroups of patients. In this context, further studies involving a larger number of patients are needed to confirm the study results.

## 5. Conclusions

The EMG recordings showed that in comparison to non-TMD cleft patients, children diagnosed with CLP and pain-related TMD have greater temporalis and masseter muscle activity at rest, reduced temporalis muscle activity during maximum voluntary contraction, and a higher Asymmetry Index for the temporalis and masseter muscles in the rest position. The altered masticatory muscle activity in TMD-P children can affect their muscle function.

## Figures and Tables

**Table 1:** The distribution of TMD-pain subjects according to RDC/TMD diagnosis.

Diagnosis	TMD-pain group (*n*=31)	
	*n*	%
Myofascial pain without limited mouth opening (Group Ia)	12	38.7
Myofascial pain with limited mouth opening (Group Ib)	3	9.7
Arthralgia (Group IIIa)	12	38.7
Group Ia and IIIa	3	9.7
Group Ib and IIIa	1	3.2

**Table 2:** The occlusal characteristics in the children studied.

Variable		TMD-pain group		Non-TMD group	
		*n*	%	*n*	%
Vertical overlap	Normal^1^	12	38.7	19	59.4
	Increased^2^	7	22.6	7	21.9
	Absence^3^	12	38.7	6	18.7
Overjet	Normal^4^	8	25.8	13	40.6
	Increased^5^	7	22.6	7	21.9
	Anterior crossbite	17	54.8	13	40.6
Posterior crossbite	No	7	22.6	12	37.5
	Yes	24	77.4	20	62.5
Angle class	I	10	32.3	17	53.1
	II	7	22.6	5	15.6
	III	14	45.2	10	31.2
Lateral open bite	No	22	71.0	24	75.0
	Yes	9	29.0	8	25.0

^1^Upper incisors is one-third of the clinical crown of lower incisors; ^2^categorized as ≥3 mm; ^3^the overlap absence (anterior open bite); ^4^upper central incisors did not exceed 3 mm; ^5^categorized as ≥3 mm.

**Table 3:** The results of the repeatability of the recording protocol.

Region	Activity	1 examination		2 examination		*P* value
		Mean	SD	Mean	SD	
Temporalis muscles	Rest	6.06	2.12	6.21	2.07	0.916
	MVC	109.63	41.01	109.98	41.09	0.994
Masseter muscles	Rest	5.25	2.10	5.41	2.14	0.948
	MVC	107.41	34.29	107.82	34.22	0.993

**Table 4:** Electrical activity of the masticatory muscles at clinical mandibular rest position in the children studied.

Region	Variable	Gender	TMD-pain group			Non-TMD group			*P* value
			*n*	Mean	SD	*n*	Mean	SD	
Temporalis muscles	Electrical activity (*μ*V/*μ*V%)	Females	15	6.79	1.43	14	5.80	1.24	0.0554
		Males	16	7.53	2.43	18	6.06	1.95	0.0596
		Total	31	7.17	2.01	32	5.94	1.66	0.0102^∗^
	Asymmetry index (%)	Females	15	15.81	7.66	14	10.19	4.97	0.1557
		Males	16	16.13	7.62	18	9.94	4.68	0.0807
		Total	31	15.99	7.02	32	10.06	4.33	0.0218^∗^
Masseter muscles	Electrical activity (*μ*V/*μ*V%)	Females	15	5.18	2.37	14	3.98	1.97	0.1516
		Males	16	6.08	2.11	18	4.64	2.01	0.0509
		Total	31	5.64	2.25	32	4.35	1.98	0.0188^∗^
	Asymmetry index (%)	Females	15	14.35	7.01	14	5.53	2.44	0.0080^∗^
		Males	16	12.04	6.67	18	6.08	3.42	0.0478^∗^
		Total	31	13.25	6.69	32	5.81	2.46	0.0010^∗^

^∗^Statistically significant difference.

**Table 5:** Electrical activity of the masticatory muscles at maximal voluntary contraction (MVC) in the children studied.

Region	Variable	Gender	TMD-pain group			Non-TMD group			*P* value
			*n*	Mean	SD	*n*	Mean	SD	
Temporalis muscles	Electrical activity (*μ*V/*μ*V%)	Females	15	99.83	37.05	14	110.55	40.04	0.4607
		Males	16	102.98	26.68	18	129.78	48.20	0.0576
		Total	31	101.46	31.61	32	121.37	45.17	0.0477^∗^
	Asymmetry index (%)	Females	15	15.93	7.30	14	7.53	3.03	0.0607
		Males	16	5.81	2.48	18	5.91	2.87	0.9563
		Total	31	10.71	5.21	32	6.62	2.93	0.0858
Masseter muscles	Electrical activity (*μ*V/*μ*V%)	Females	15	98.24	31.53	14	102.56	31.33	0.7144
		Males	16	103.01	35.33	18	115.29	41.76	0.3649
		Total	31	100.70	33.08	32	109.72	37.54	0.3163
	Asymmetry index (%)	Females	15	15.31	7.32	14	8.09	4.85	0.0330^∗^
		Males	16	6.75	3.34	18	6.59	3.38	0.9418
		Total	31	10.89	5.59	32	7.24	3.50	0.0773

^∗^Statistically significant difference.
